# Gender-related differences in the association of serum levels of vitamin D with body mass index in northern Iranian population: the PERSIAN Guilan Cohort Study (PGCS)

**DOI:** 10.1186/s40795-022-00637-1

**Published:** 2022-12-08

**Authors:** Farahnaz Joukar, Mehrnaz Asgharnezhad, Mohammadreza Naghipour, Kourosh Mojtahedi, Arsalan Salari, Alireza Mansour-Ghanaei, Fariborz Mansour-Ghanaei

**Affiliations:** 1 Gastrointestinal and Liver Diseases Research Center, Guilan University of Medical Sciences, Razi Hospital, Sardar-Jangle Ave, Rasht, 41448-95655 Iran; 2 Caspian Digestive Diseases Research Center and GI Cancer Screening and Prevention Research Center, Guilan University of Medical Sciences, Rasht, Iran; 3 Cardiovascular Diseases Research Center, Department of Cardiology, Heshmat Hospital, School of Medicine, Guilan University of Medical Sciences, Rasht, Iran; 4 Caspian Digestive Diseases Research Center, Guilan University of Medical Sciences, Rasht, Iran

**Keywords:** Vitamin D, Body Mass Index, Gender

## Abstract

**Background:**

Vitamin D (VD) insufficiency has now become a global problem throughout the world. The association between increasing body mass index (BMI) and VD insufficiency has attracted great attention in recent researches. The aim was to study if BMI was independently associated with serum 25-hydroxy VD in a large population-based study, specify by gender.

**Methods:**

Data on 9520 adults 35 years and older participating in PERSIAN Guilan Cohort Study (PGCS) were used. Serum levels of 25-hydroxy VD less than 20 ng/mL (50 nmol/L**)** was used as a measure of VD inadequacy. Multiple logistic and linear regression analyses were used to estimate the strength of the association between VD and BMI before and after adjusting for demographic factors and lifestyle variables.

**Results:**

After adjustment in male population, overweight (adjusted OR = 1.2, *P* < 0.05) and obese (adjusted OR = 1.4, *P* > 0.05) individuals were more likely to have VD inadequacy than normal weight counterparts. Moreover, there was a weak inverse linear association between BMI and serum 25 (OH) D levels in males (β = -0.14, *P* value > 0.05). In contrast, no significant associations between BMI and serum 25 (OH (D levels were observed in females. In male population, higher BMI were associated with lower serum vitamin 25 (OH) D levels.

**Conclusions:**

However, association between BMI and VD level was not observed in female population. The suggestion of current study for public health was special consideration to serum VD levels in over weight and obese males.

## Background

Vitamin D (VD) insufficiency has now become a global problem throughout the world [1] and is specifically a challenge in Iran [2]. VD insufficiency affects about half of the Iranian population [2]. On the other hand, in the past decades we have witnessed a steady increase in the prevalence of obesity [3, 4]. Approximately one-third of the Iranian adult populations were overweight and obese [3, 5].

The association between increasing body mass index (BMI) and VD insufficiency has attracted great attention in recent researches [6, 7]. Findings from previous studies regarding the association of serum VD levels with obesity are conflicting [7]. While some randomized controlled clinical trials (RCTs) have linked VD supplementation to accelerate weight [8, 9], others have failed to reach such findings [10, 11]. These cross-study inconsistencies on association of VD and weight are more obvious in cross-sectional studies [7]. Some studies have shown negative correlations [12, 13], whereas others did not [14, 15].

Although the mechanism of increased risk of VD deficiency in obesity is not well understood and the causality of association has not yet been established, at the biochemical and physiological level active VD can reduce the risks of metabolic disorders and tissue damage related to adiposity [16]. As serum levels of VD are affected by latitude and longitude due to sun exposure and the climate difference [17], also, because of different genetic and cultural factors and obesity patterns [18], it seems that the association between BMI and serum levels of VD is different by region [7]. It was also mentioned in some studies that the level of VD is different according to gender [19–21].

Globally, extreme obesity is more prevalent among women than men, with up to 70% of extremely obese persons being women [22]. Women have relatively more body fat than men and store more fat in the gluteal–femoral region, while men typically store more fat in the visceral (abdominal) depot [6, 23]. Some studies have found a higher prevalence of VD deficiency among men than women [24, 25]. As VD is a fat soluble vitamin that may potentially be sequestrated in adipose tissue [14], one could hypothesize that a gender difference in the prevalence of VD deficiency is related to differences in the amount of body fat and/or its distribution. To address this hypothesis, we study the relationships between VD status, BMI and gender.

However, the association of VD and BMI has been explored in previous studies, but the results are conflicting may be due to small sample sizes, study populations heterogeneity, and methodological differences. Thus, the present study was conducted to determine if the associations between BMI and VD could be found in a large population-based study based on data from the PERSIAN Guilan Cohort Study (PGCS), a prospective, population-based cohort study in Guilan, Iran.

## Methods

### Recruitment and study design

Data from the PERSIAN Guilan Cohort Study (PGCS) were used for this community based cross-sectional study [26]. This data was collected between October 8, 2014, and January 20, 2017. The PERSIAN (Prospective Epidemiological Research Studies in Iran) study [27, 28], assessed the health status of Iranian population, to determine the incidence, prevalence and risk factors of non-communicable diseases.

The sampling design and methods used have been previously reported elsewhere in detail [26, 28]. In total, 10,520 participants (aged 35–70 years) included in this study. For analytic purposes, we excluded participants without information on VD (*n* = 1000), yielding a final sample of 9520. The participation rate was 83.2% [26]. The regional Ethics Committee approved the study protocol and written informed consent was obtained from each participant [26].

### Data collection

VD, which is produced in the skin or taken from the dietary sources, is hydroxylated to 25(OH) D. Therefore, Serum 25(OH) D levels have been used as a biomarker for VD adequacy [29]. Blood sample collection and measurement of Serum 25(OH) D methods have been previously reported elsewhere in detail. Serum 25-Hydroxyl VD was determined by using a commercially available electrochemiluminescence immunoassay with Roche Elecsys 2010 and Cobas E411 auto analyzer (Roche Diagnostics GmbH, Mannheim, Germany) [30]. Consistent with the latest update of VD status definition [31], we categorized serum 25(OH) D levels as less than 12 ng/mL (30 nmol/L) (deficiency), 12 to 20 ng/mL (50 nmol/L) (insufficiency)), and 20 ng/mL (50 nmol/L) or more (sufficiency).

BMI was calculated by the use of measured weight (in kg) and height (in m) according to National Health and Nutrition Examination Survey Manual [32]. BMI was classified as underweight (BMI < 18.5 kg/m2), normal weight (BMI = 18.5–24.99 kg/m2), overweight (BMI = 25–29.9 kg/m2) and obese (BMI ≥ 30 kg/m2) according to the Centers for Disease Control and Prevention recommendation [33]. Other measurements of interest for analysis as potential confounders were demographic factors (age, sex, residency and educational level), and lifestyle characteristics (physical activity, alcohol consumption and smoking). The details of the evaluation and classification of these measurements have been previously described [26, 30].

### Statistical analyses

The statistical analyses were conducted using SPSS version 16.0 (SPSS Inc., Chicago, IL, USA). Descriptive statistics for characteristics of the participants were calculated for the total population and according to the VD status (sufficient (≥ 20 ng/mL), inadequacy (< 20 ng/mL)) and BMI categories. Group comparisons were performed with independent student t-test and chi-square tests and analysis of variance (ANOVA). Multivariate linear and logistic regression analysis was performed and models were created to assess the linear and nonlinear association of VD with BMI. By using serum VD levels as a dependent variable, we assessed the independent effect of BMI with adjustment for demographic variables (age, sex, residency and educational level) and lifestyle characteristics (physical activity, smoking, and alcohol consumption). Adjusted odd ratios with 95% confidence intervals were reported. In addition, all regression analyses were stratified by sex. Results were considered statistically significant for *P*-value of less than 0.05. The sensitivity analysis was performed again after removing the people who consumed VD.

## Results

Baseline characteristics of the participant overall and by serum 25(OH) D levels are shown in Table 1. VD sufficiency (25(OH) D levels ≥ 20 ng/mL (50 nmol/L)) was found in almost half (50.4%) of the study population.

**Table 1:** Baseline characteristics of study population overall and by serum 25-Hydroxy vitamin D levels

Variables	Serum 25-Hydroxy Vitamin D level < 20 ng/mL (50 nmol/L))	Serum 25-Hydroxy Vitamin D level ≥ 20 ng/mL (50 nmol/L)	*P*_value*	total
**Number of participants**	4719(49.6%)	4801(50.4%)		9520
**Serum 25(OH)D (nmol /L), mean ± SD**	**12.2 ± 4.4**	**31.1 ± 10.42**	< 0.001	21.75 ± 12.3
**Age (year), mean ± SD**	**50.1 ± 8.5**	**52.7 ± 9.07**	< 0.001	51.45 ± 8.9
**Residency, N (%)**				
**Urban**	2578(45.2%)	2223(46.3%)	0.1	4354(45.7%)
**Education, N (%)**				
**High school or less**	858(50.3%)	1844(47.6%)	0.08	4424(48%)
**Gender, N (%)**				
**Male**	**2143(45.4%)**	**2281(47.5%)**	0.02	3809(46.5%)
**BMI (kg /m)2, mean ± SD**	**28.3 ± 5.1**	**27.9 ± 5.05**	0.001	28.15 ± 5.1
**Physical activity** **(**METhrs /day**), mean ± SD**	**40.6 ± 8.6**	**41.2 ± 8.8**	0.002	40.9 ± 8.7
**Use of Alcohol, N (%)**	669(14.4%)	733(15.3%)	0.1	1402(14.7%)
**Smoking state, N (%)**				
**Current smoker**	**1099(23.3%)**	**1247(26%)**	0.006	2346(24.6%)
**Receiving Vitamin D supplementation** ^a^**, N (%)**	377(8%)	343(7.1%)	0.06	420(7.6%)

*Abbreviations: 25(OH) D* 25-hydroxyvitamin D, *BMI* Body mass index, *METs* Metabolic equivalent rates

^a^ Vitamin D supplements use of at least monthly

^*^ Statistical significance based on Chi-square test for categorical variables or the independent T test for continuous variables

VD sufficient participants were significantly older, more likely to be men and smoker, had a lower BMI, and were more physically active compared with VD inadequate participants (all *P* < 0.05) (Table 1).

The most of study population was distributed across normal weight (27.7%), overweight (39.7%), and obese (32.6%) (Table 2). Normal weight participants were significantly older, more likely to be men, smoker, alcohol consumer, lived in urban area, and were more physically active compared with overweight and obese participants (all *P* < 0.05) (Table 2).

**Table 2:** Baseline characteristics of study population by BMI categories

Variables	BMI categories, kg/m2			
	< 25 (normal)	25 to < 30 (overweight)	≥ 30 (Obese)	P_ value*
**Number of participants (total = 9520)**	2634(27.7%)	3782(39.7%)	3104(32.6%)	
**Vitamin D status, N (%)**				
**Sufficiency (25 OHD ≥ 20 ng/mL)**	**1392(52.8%)**	**1900(50.2%)**	**1509(48.6%)**	0.006
**Serum 25(OH)D (nmol /L), mean ± SD**	**22.41 ± 12.3**	**21.76 ± 12.3**	**21.18 ± 12.3**	0.001
**BMI (kg /m)2, mean ± SD**	**22.39 ± 2.0**	**27.47 ± 1.4**	**33.8 ± 3.6**	< 0.001
**Age (year), mean ± SD**	**52.14 ± 9.2**	**51.35 ± 8.8**	**51 ± 8.6**	< 0.001
**Residency, N (%)**				
**Urban**	**1289(48.9%)**	**1672(44.2%)**	**1393(44.9%)**	< 0.001
**Gender, N (%)**				
**Male**	**1837(69.7%)**	**1881(49.7%)**	**706(22.7%)**	< 0.001
**Education, N (%)**				
**High school or less**	1075(49.5%)	1531(48.1%)	1203(46.6%)	0.131
**Physical activity** **(**METhrs /day**), mean ± SD**	**43.1 ± 9.5**	**41 ± 8.8**	**39.1 ± 7.3**	< 0.001
**Use of Alcohol, N (%)**	**579(22.0%)**	**582(15.4%)**	**241(7.8%)**	< 0.001
**Smoking state, N (%)**				
**Current smoker**	**1073(40.7%)**	**909(24%)**	**364(11.7%)**	< 0.001
**Receiving Vitamin D supplementation **^a^**, N (%)**	216(8.2%)	274(7.2%)	230(7.4%)	0.336

*Abbreviations: 25(OH) D* 25-hydroxyvitamin D, *BMI* Body mass index, *METs* Metabolic equivalent rates

^a^ Vitamin D supplements use of at least monthly

^*^ Statistical significance based on Chi-square test for categorical variables or the independent T test for continuous variables

**Fig. 1:**
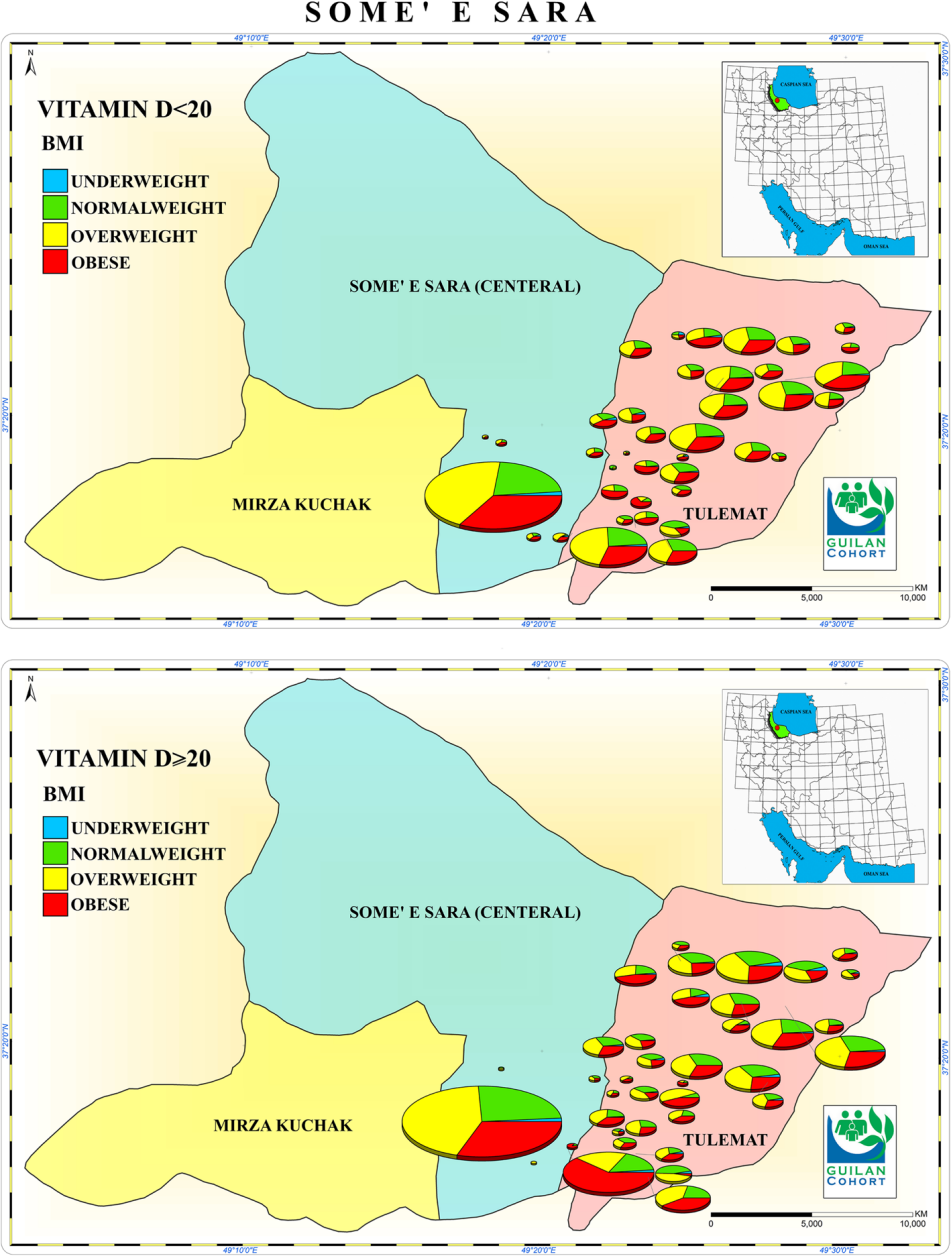
Geographical location of participants with serum 25OHD levels concentration ≤ 20 ng/Ml and ≥ 20 ng/Ml) and BMI

Geographical location using Garmin GPSMAP 78 s of participants that had a 25(OH)-D level concentration ≤ 20 ng/mL and ≥ 20 ng/mL and BMI are shown in Fig. 1.

The evaluation of the linear associations between BMI and Serum 25(OH) D levels by multiple linear regression analyses shown in total population, higher BMI were associated with lower serum 25(OH) D levels, but this association disappeared after fully adjustment for demographic and lifestyle variables and VD supplementation (Table 3). The evaluation of the associations between BMI and Serum 25(OH) D levels in females shown BMI were not statistically significantly associated with serum 25(OH) D levels, unadjusted and after adjustment for potential confounders (Table 3).

**Table 3:** Linear regression analysis of BMI associated with serum 25-hydroxyvitamin D stratified by gender

**BMI (kg /m)2**	**Serum 25-hydroxyvitamin D (nmol/L)**								
	**all**			**male**			**female**		
	**Regression coefficients (B)**	**95% confidence interval**	***P*-value**	**Regression coefficients (B)**	**95% confidence interval**	***P*-value**	**Regression coefficients (B)**	**95% confidence interval**	***P-value***
Model 1	-0.09	-0.1 to -0.04	< 0.001	-0.19	-0.2 to-0.1	< 0.001	-0.06	-0.1 to 0.01	0.09
Model 2	-0.06	-0.1 to -0.006	0.03	-0.16	-0.2 to -0.07	< 0.001	-0.003	-0.08 to 0.07	0.9
Model 3	-0.05	-0.1 to 0	0.05	**-0.14**	**-0.2 to -0.06**	**0.001**	-0.005	-0.08 to 0.07	0.8

Model 1: crude model. Model 2: model 1 adjusted for age, sex, residency and educational level. Model 3: model 2 additionally adjusted for physical activity (METhrs /day), alcohol use, and smoking and Vitamin D supplementation

In contrast, in males higher BMI were associated with lower Serum 25(OH) D levels, and this association remained statistically significant after fully adjustment for potential confounders.

Fully adjusted regression coefficients for BMI in relation to 25(OH) D in males were -0.14 (*P* < 0.05), it means an increase in males BMI of 1 kg/m2 was associated with a 0.14 decrease in nmol/L serum 25(OH) D level (Table 3).

Logistic regression analyses were performed to evaluate the non-linear association between BMI categories and VD status (Table 4). The crude and adjusted odds ratio (OR) for VD inadequacy (serum 25(OH) D level < 20 ng/mL (50 nmol/L)) in relation to BMI categories was shown in Table 4. There were no independent associations between BMI categories and VD status after controlling for demographic and lifestyle factors (*P* > 0.05). In logistic regression analyses stratified for sex, similar results were observed for females but not male, which indicates that overweight and obese males had a higher risk of VD inadequacy compared with normal weights (adjusted OR = 1.2, *P* < 0.05; adjusted OR = 1.4, *P* < 0.05, respectively) (Table 4). The sensitivity analysis was performed again after removing the people who consumed VD, and no significant difference was obtained with the initial results.

**Table 4:** Crude and adjusted Odds ratios for serum 25 OHD levels < 20 ng/mL (50 nmol/L)) according to BMI categories stratified by gender

sex	**BMI categories, kg/m2**	**Vitamin D inadequacy( serum 25 OHD levels < 20 ng/mL (50 nmol/L))**					
		**Crude Odds ratio**	**95% confidence interval**	*P*-value*	**Adjusted Odds ratio**^a^	**95% confidence interval**	P-value*
all	** < 25(ref)**						
	**25 to < 30**	**1.1**	**1.1–1.2**	**0.040**	1.1	0.9–1.2	0.2
	** ≥ 30**	**1.2**	**1.1–1.4**	**0.001**	0..93	0.9–1.2	0.2
male	** < 25(ref)**						
	**25 to < 30**	**1.1**	**1.1–1.4**	**0.013**	**1.2**	**1.1–1.4**	**0.01**
	** ≥ 30**	**1.4**	**1.4–2**	** < 0.001**	**1.4**	**1.2–1.6**	**0.001**
female	** < 25(ref)**						
	**25 to < 30**	0.9	0.8–1.2	0.850	1.1	0.9–1.3	0.160
	** ≥ 30**	0.9	0.8–1.2	0.670	1.1	0.9–1.4	0.065

^a^ Adjusted for age, sex, residency and educational level,physical activity (METhrs /day), alcohol use, and smoking and Vitamin D supplementation

^*^ Statistical significance based on logistic regression analyses

## Discussion

Various factors affect vitamin D levels in the body. The present study examined the vitamin D status of participants in PGCS and analyzed the effect of gender on vitamin D status. The principal finding in this study is that obese men had higher odds of VD deficiency than obese women even after adjustment for the confounders. The connection between reduced VD concentrations and obesity is well documented. The results of our study are consistent with previous studies in other populations that have found negative associations between 25(OH) D and BMI [6, 34–36]. The studies have found a higher prevalence of VD deficiency among men than women [6, 35]. The high prevalence of VD deficiency in obese individuals is most likely due to volumetric dilution in the larger volumes of fat, serum, liver, and muscle present in obese individuals. However, other mechanisms cannot be completely ruled out as they may contribute concomitantly. A low VD concentration cannot yet be excluded as a cause of obesity due to the incompletely explored effects of VD receptors found in adipose tissue [37]. Consistent with this, previous studies have shown that VD deficiency in obese individuals is caused by reduced bioavailability resulting from VD deposition in body fat compartments [23], particularly in visceral fat stores [38].

A study showed that there is an association between BMI and serum 25(OH) D levels in adults, excluding women living in developing countries. The results indicated that in women, the association between BMI and serum 25(OH) D levels differs between developed and developing countries. They proposed that gender, ethnicity, and age may play a role in mediating the association of BMI and VD status [7]. The reason for gender differences in the association of VD serum levels with BMI is not yet clear, it may be due to the VD-binding protein. The relationships between obesity and VD-binding protein were hypothesized to be positive in females and negative in males [39]. Differences in body composition and less body fat in males than females with the same BMI may be another reason [40, 41].

The main source of VD in the human population is exposure of the skin to ultraviolet B (UVB) radiation (290,315 nm) [42–44]. It is possible that obese individuals have less skin exposure to the sun than non-obese individuals, resulting in decreased VD synthesis [42].

On the other hand, two British studies confirmed that sun exposure does not vary between normal weight and obese people [43, 44]. Another study reported that cutaneous VD synthesis upon UVB exposure is similar in people with different body mass index (BMI) values ​​[23]. The findings from the ANSAViD Cohort Study revealed VD deficiency in obese woman is not related to reduced sun exposure [44]. Especially in developing country like IRAN, the difference in vitamin levels in men and women could also be explained by different clothing style, which is related to religion or culture [7]. In our study, women had more VD than men, despite their clothing style. The lifestyle differences between males and females could provide another explanation for the gender differences in serum VD levels suggested by Yan et al. were given in Chinese people [45]. In our region, especially in rural areas, people generally are farmer and women work in the fields so they spend more time in outdoor in the daytime, which directly affects their sun exposure and VD synthesis.

Therefore, the BMI alone cannot be a decisive indicator for the reduction of VD in obese men. This is consistent with other studies where, despite the fact that BMI reflects total body fat, BMI is not a coordinate of body composition and may not be as relevant to younger populations, which for the most part have higher oblique mass than older adults. In addition, it is important to recognize that indeed individuals of normal weight may have excess visceral fat, increasing their risk of many chronic diseases [46]. Also, analysts from the Perelman School of Pharmaceutical, College of Pennsylvania say that BMI does not take into account muscle mass, bone thickness, general body composition, and racial and gender differences [47]. Thus, there are many possible explanations for the inverse association between increased obesity, especially abdominal obesity, and low plasma VD concentrations, but so far none of these hypotheses can fully elucidate this association. Therefore, it is proposed to study other anthropometric indicators such as WHR and several mechanisms have an impact on the interaction between VD and obesity. Therefore, further studies are needed to investigate this.

## Limitation

In various studies [48, 49], the dosage or duration of VD consumption was mentioned, but it is a limitation of the present study that the consumption of VD in this study was only in the form of eating or not eating, and the dose was not recorded.

## Conclusions

In conclusion, the present study findings revealed that in male population, higher BMI were associated with lower serum vitamin 25 (OH) D levels and overweight and obese males were more likely to be VD inadequate (serum 25(OH) D levels < 20 ng/mL) than their normal weight counterparts. However, no associations between BMI and serum 25(OH) D levels were observed in female population. The possible public health proposal of the current study was a special consideration of serum VD levels in overweight and obese men. Therefore, future guidelines for monitoring VD concentrations should not only take into account obesity, but also gender.

## Data Availability

The datasets obtained during this study will be available upon request to the corresponding author.

## Abbreviations

VD: Vitamin D
BMI: Body Mass Index
25(OH) D: 25-Hydroxyvitamin D
PGCS: PERSIAN Guilan Cohort Study
OR: Odd ratio
RCTs: Randomized controlled clinical trials
